# Comparative transcriptome analysis of cotton fiber development of Upland cotton (*Gossypium hirsutum*) and Chromosome Segment Substitution Lines from *G. hirsutum × G. barbadense*

**DOI:** 10.1186/s12864-017-4077-8

**Published:** 2017-09-08

**Authors:** Peng-tao Li, Mi Wang, Quan-wei Lu, Qun Ge, Md. Harun or Rashid, Ai-ying Liu, Ju-wu Gong, Hai-hong Shang, Wan-kui Gong, Jun-wen Li, Wei-wu Song, Li-xue Guo, Wei Su, Shao-qi Li, Xiao-ping Guo, Yu-zhen Shi, You-lu Yuan

**Affiliations:** 1 State Key Laboratory of Cotton Biology, Key Laboratory of Biologiacl and Genetic Breeding of Cotton, The Ministry of Agriculture, Institute of Cotton Research, Chinese Academy of Agricultural Science, Anyang, Henan 455000 China; 2 National Key Laboratory of Crop Genetic Improvement, Huazhong Agricultural University, Wuhan, Hubei 430070 China; 3 College of Agriculture, Yangtze University, Jingzhou, Hubei 434025 China

**Keywords:** *Gossypium hirsutum*, *G. hirsutum* × *G. barbadense*, Chromosome Segment Substitution Lines, Transcriptome analysis, Fiber development

## Abstract

**Background:**

How to develop new cotton varieties possessing high yield traits of Upland cotton and superior fiber quality traits of Sea Island cotton remains a key task for cotton breeders and researchers. While multiple attempts bring in little significant progresses, the development of Chromosome Segment Substitution Lines (CSSLs) from *Gossypium barbadense* in *G. hirsutum* background provided ideal materials for aforementioned breeding purposes in upland cotton improvement. Based on the excellent fiber performance and relatively clear chromosome substitution segments information identified by Simple Sequence Repeat (SSR) markers, two CSSLs, MBI9915 and MBI9749, together with the recurrent parent CCRI36 were chosen to conduct transcriptome sequencing during the development stages of fiber elongation and Secondary Cell Wall (SCW) synthesis (from 10DPA and 28DPA), aiming at revealing the mechanism of fiber development and the potential contribution of chromosome substitution segments from Sea Island cotton to fiber development of Upland cotton.

**Results:**

In total, 15 RNA-seq libraries were constructed and sequenced separately, generating 705.433 million clean reads with mean GC content of 45.13% and average Q30 of 90.26%. Through multiple comparisons between libraries, 1801 differentially expressed genes (DEGs) were identified, of which the 902 up-regulated DEGs were mainly involved in cell wall organization and response to oxidative stress and auxin, while the 898 down-regulated ones participated in translation, regulation of transcription, DNA-templated and cytoplasmic translation based on GO annotation and KEGG enrichment analysis. Subsequently, STEM software was performed to explicate the temporal expression pattern of DEGs. Two peroxidases and four flavonoid pathway-related genes were identified in the “oxidation-reduction process”, which could play a role in fiber development and quality formation. Finally, the reliability of RNA-seq data was validated by quantitative real-time PCR of randomly selected 20 genes.

**Conclusions:**

The present report focuses on the similarities and differences of transcriptome profiles between the two CSSLs and the recurrent parent CCRI36 and provides novel insights into the molecular mechanism of fiber development, and into further exploration of the feasible contribution of *G. barbadense* substitution segments to fiber quality formation, which will lay solid foundation for simultaneously improving fiber yield and quality of upland cotton through CSSLs.

**Electronic supplementary material:**

The online version of this article (10.1186/s12864-017-4077-8) contains supplementary material, which is available to authorized users.

## Background

On the global scale, cotton is not only one of the major crops, but also the most important plant producing natural fibers for the textile industry. Being composed of 46 diploid (2n = 2× = 26) and 5 allotetraploid (2n = 4× = 52) species [1], only 4 species of *Gossypium* were cultivated worldwide, namely *G. herbaceum*, *G. arboreum*, *G. hirsutum* and *G. barbadense*. The allotetraploid cotton species, Upland cotton (*G. hirsutum*) and Sea Island cotton (*G. barbadense*), derived from a natural hybridization event between A-genome and D-genome 1–2 million years ago [2], contribute over 95% of cotton fiber yield. Upland cotton possesses the characteristics of high yield and moderate fiber quality, while Sea Island cotton is famous as its premium fiber quality performance and low fiber productivity. Facing the diminishing of arable land while increasing of human population, to develop novel varieties combining high productivity of *G. hirsutum* and excellent fiber quality of *G. barbadense* remains a preferential alternative in cotton breeding [3]. Chromosome segment Substitution lines (CSSLs), of which the genome was composed mostly of the recipient parent and minimal chromosome segments substituted from the donor parent, provided useful tools to take full advantages of both Upland and Sea Island cotton through marker assisted-selection (MAS) and conventional breeding procedures such as hybridization, backcross and selfing. To provide ideal materials for further genome research and crop improvement through MAS, CSSLs have been intensively applied to Quantitative Trait Locus (QTL) mapping for yield, quality, disease resistance and stress tolerance in a plenty of crops such as cotton [4–13], tomato (*Lycopersicon esculentum*), rice (*Oryza sativa*) and wheat (*Triticum aestivum*) [14–17].

Cotton fiber, a single-celled seed trichome developed from ovule epidermal cells, is an ideal model for investigating the mechanism of cell elongation, cell wall, and cellulose biosynthesis, which undergoes four overlapping developmental stages: initiation (−3 to +3 days post anthesis, DPA), elongation (3 to 23 DPA), secondary cell wall synthesis (16 to 40 DPA) and maturation (40 to 50 DPA) [18–22]. Fiber quality traits were collectively determined by performance of each of the aforementioned stages, so any disorders in these stages will significantly impact the final formation of the quality traits of fiber length, micronaire, strength, elongation and uniformity [23]. Along with the rapid development of high-throughput sequencing technologies, the genome sequencing of *G. raimondii* [24], *G. arboretum* [25], *G. hirsutum* [26, 27] and *G. barbadense* [28, 29] have been reported, which doubtlessly enhanced the cognition of polyploid properties of *Gossypium* species, including size variation of functional genome and significance of agronomic traits, and would further promote the research activities of structural genetics and functional genomics. As an effective tool to reveal the molecular mechanism of particular biological processes through concentrating on the overall level of gene expression and regulation, transcriptome analysis provided a novel high-throughput method for comprehending fiber growth system in combination with gene expression comparison between the different cotton species or between diverse developmental stages [30–35]. As a complicated biological process, fiber development was regulated by plant hormones such as auxin [36, 37], gibberellins [38], brassinosteroid [39], ethylene [40, 41], abscisic acid [42, 43] and cytokinin [44, 45]. Furthermore, carbohydrate, lipid, lignin, flavonoid and phenylalanine metabolisms have also been proved to play significant roles in fiber development [46–50] and some related genes are identified, such as *GhKCS13* [51], *GbEXPA2* [52], *GbEXPATR* [53] and so on. Despite all the above-mentioned results, the molecular mechanism of fiber elongation and Secondary Cell Wall (SCW) formation remains unclear, making it necessary for further investigations.

In the present study, two CSSLs, MBI9915 and MBI9749, having some substituted *G. barbadense* chromosome segments, were selected to conduct transcriptome analysis owing to their excellent fiber performance, and the recurrent *G. hirsutum* parent CCRI36 was also sequenced as the genome background and low fiber quality control. The CSSLs were developed from several generations of backcrossing and selfing of a cross between CCRI36, a *G. hirsutum* cultivar, and Hai 1, a *G. barbadense* cultivar, with *G. hirsutum* as the recurrent parent. Two of the most important fiber properties, fiber length and strength, were formed largely on the process of fiber elongation and SCW biosynthesis, respectively [54], therefore, the developing fibers were sampled at 10, 15, 20, 25 and 28 DPA for transcriptome sequencing and analysis. Aiming at exploring the differentially expressed genes (DEGs) relevant to fiber growth and inquiring into the possible contribution of chromosome substitution segments from Sea Island cotton to fiber quality formation, multiple comparisons among the RNA-seq data from different fiber developing stages of the three materials (two CSSLs and CCRI36) were performed. Plenty of DEGs were identified based on GFOLD analysis. The DEGs were then underwent GO functional enrichment and KEGG metabolic pathway analysis followed by accuracy validation using quantitative real-time PCR (qRT-PCR). The results indicated that the candidate genes bound up with fiber formation would contribute to the comprehension of the mechanism of cotton fiber elongation and SCW synthesis, and further make a significant difference in the cotton breeding and genomics research.

## Results and Discussion

### The phenotypic traits and substitution background of CSSLs

In the study, the descriptive statistic characteristics of fiber yield and quality traits of MBI9915, MBI9749, the recurrent parent CCRI36, and the donor parent Hai 1 were shown in Table 1. With regard to the fiber quality performance, fiber length and strength of the two CSSLs were superior to those of CCRI36. In contrast, the boll weight and lint percentage of the two CSSLs were better than those of Hai 1, indicating that the two CSSLs showed a significant improvement in fiber yield as compared to the donor parent. The two CSSLs, which had significant fiber quality performance, would provide excellent materials for the further research of fiber development and functional gene identification.

**Table 1:** Fiber yield and quality traits performance of two CSSLs, CCRI36 and Hai 1 at Anyang in 2014

Materials	FL(mm)	FU(%)	FM(unit)	FS(cN/tex)	FE(%)	BW(g/boll)	LP(%)
CCRI36	29.28	85.21	4.49	31.29	6.99	5.48	39.47
Hai 1	35.53	83.3	4.21	37.7	6	2.7	30.9
MBI9749	30.42	85.87	4.28	33.75	7.15	5.47	38.14
MBI9915	32.08	86.05	4.182	35.78	7.05	5.3603	39.895

*FL* Fiber Length, *FU* Fiber Uniformity, *FM* Fiber Micronaire, *FS* Fiber Strength, *FE* Fiber Elongation, *BW* Boll weight, *LP* Lint Percentage

In the high-density linkage map of CSSLs population derived from CCRI36 × Hai 1 which was described previously [12], 527 Simple Sequence Repeat (SSR) markers were selected at a marker interval of approximately one SSR marker per 10 cM genetic distance to identify chromosome substitution segments in the two CSSLs. The results showed that there were 19 substituted segments from *G. barbadense* and 97.90% genomic background recovery to *G. hirsutum* in MBI9915, while 19 substituted segments and 96.10% background recovery in MBI9749 (Table 2). Heterozygocity of the substituted segments in the two CSSLs were analyzed (Table 2) and the results indicated that there was less than 4% of the total substituted segments of *G. barbadense* in the two CSSLs were identified, which indicated that the CSSLs were eligible for our intended studying of transcriptome analysis for functional dissection of the substituted segments on fiber quality formation.

**Table 2:** Genetic background of the two CSSLs

Materials	Background recovery percentage	Introgressive segment number	Introgressive segment length	Homozygous segment number	Homozygous segment percentage	Heterozygous segment number	Heterozygous segment percentage
MBI9915	97.90%	19	105.5 cM	13	1.50%	6	0.60%
MBI9749	96.10%	19	194.4 cM	5	1.10%	14	2.70%

### Transcriptome sequencing and alignment to the reference genome

With the purpose of systematic identification of the critical genes affecting the cotton fiber development, 15 RNA-seq libraries of fiber samples were constructed and analyzed, which included the ones of fiber samples at 10, 15, 20, 25 and 28 DPA in MBI9915, MBI9749, and CCRI36. After removal of low-quality reads, a total of 750.433 million clean reads (approximately 88.18 Gb data) were obtained, of which the average reads per sample were 47.029 million (Table 3). In the alignment analysis, the proportion of clean data that were mapped to the genome of *G. hirsutum* reached to 91.93–94.30%, together with not less than 89.02% of the Q30 and over 44.79% of the GC content, implied a reliable quality of the RNA-seq result.

**Table 3:** Summary of the RNA-seq outcomes of 15 separately pooled samples

Samples	Total reads	Mapped reads	Uniq mapped reads	Multiple mapped reads	GC content(%)	Q30(%)
H1–10	28.874	26.955 (93.35%)	23.163 (80.22%)	3.792 (13.13%)	44.92	89.56
H1–15	46.506	43.543 (93.63%)	35.628 (76.61%)	7.914 (17.02%)	45.01	89.88
H1–20	58.818	54.118 (92.01%)	46.657 (79.33%)	7.460 (12.68%)	45.32	89.59
H1–25	74.935	69.157 (92.29%)	60.808 (81.15%)	8.349 (11.14%)	44.83	90.04
H1–28	45.842	42.186 (92.02%)	37.261 (81.28%)	4.924 (10.74%)	44.87	91.56
H2–10	41.384	39.026 (94.30%)	32.371 (78.22%)	6.656 (16.08%)	45.16	90.86
H2–15	51.063	47.661 (93.34%)	38.451 (75.30%)	9.210 (18.04%)	44.98	89.02
H2–20	47.327	44.331 (93.67%)	37.008 (78.20%)	7.323 (15.47%)	45.54	90.28
H2–25	46.285	43.048 (93.01%)	36.495 (78.85%)	6.553 (14.16%)	45.03	90.69
H2–28	46.911	43.217 (92.12%)	36.480 (77.76%)	6.737 (14.36%)	44.79	90.31
Z36–10	32.345	30.236 (93.48%)	25.116 (77.65%)	5.121 (15.83%)	44.79	89.64
Z36–15	41.266	38.850 (94.14%)	31.683 (76.78%)	7.167 (17.37%)	44.97	90.95
Z36–20	47.377	43.552 (91.93%)	36.464 (76.97%)	7.087 (14.96%)	45.26	90.27
Z36–25	47.471	43.736 (92.13%)	38.161 (80.39%)	5.575 (11.74%)	45.28	90.66
Z36–28	49.029	45.590 (92.99%)	33.337 (67.99%)	12.253 (24.99%)	46.20	90.55

H1, H2 and Z36 represent MBI 9915, MBI 9749 and CCRI36, respectively. 10 means 10 day post anthesis (DPA), 15 = 15 DPA, 20 = 20 DPA, 25 = 25 DPA and 28 = 28 DPA

**Fig. 1:**
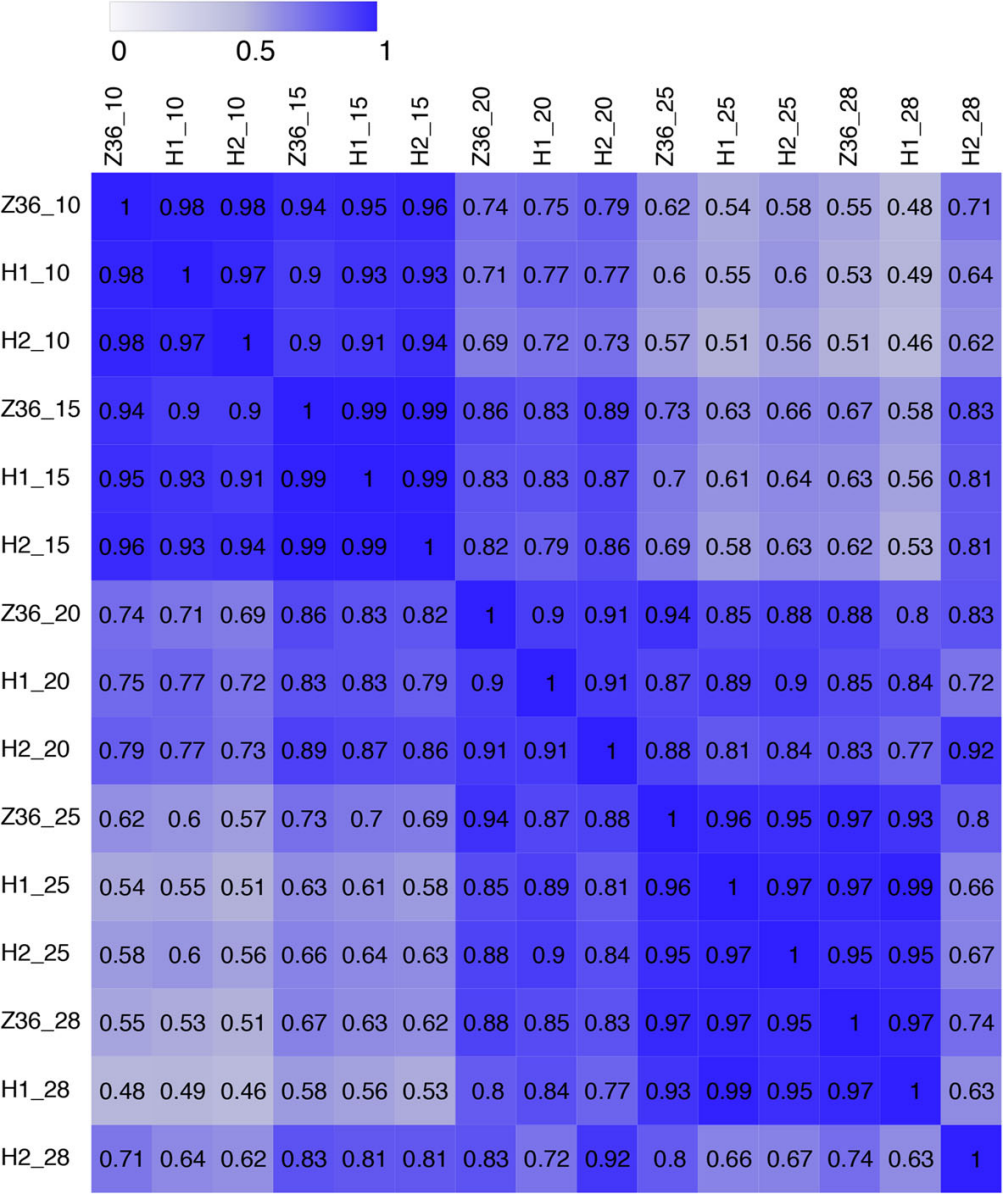
Pearson Correlation Coefficient of the genes identified from the 15 samples. H1, H2 and Z36 represent MBI9915, MBI9749 and CCRI36, respectively. 10 = 10 DPA, 15 = 15DPA, 20 = 20DPA, 25 = 25DPA, and 28 = 28DPA

A total of 10,198 genes were identified, which were expressed in no less than half of the 15 libraries. So as to test the correlations between the experimental samples, Pearson Correlation Coefficient (PCC) analysis was adopted to analyze the expression of the genes obtained from these 15 libraries (Fig. 1). The results indicated that gene expressions in all the three lines at the same stage of fiber development showed more than 90% similarities except for the genes identified between MBI9749 and MBI9915/CCRI36 at 28DPA. Such gene expression similarities were higher at both the early (10 and 15DPA) and later (25 and 28DPA) stages than at the stage of 20 DPA, which hinted a significant gene expression alteration at the stage of 20DPA during fiber development. A gradual decreasing trend was also observed in similarities between 10DPA and each of the remained later stages in all the three lines, suggesting a remarkable alteration of gene expression profiles along with the stages of fiber development. All the findings of the gene expression similarity alterations between MBI9915/MBI9749 and the recurrent parent CCRI36 might result from the distinct *G. barbadense* chromosomal segments contained in the CSSLs.

**Fig. 2:**
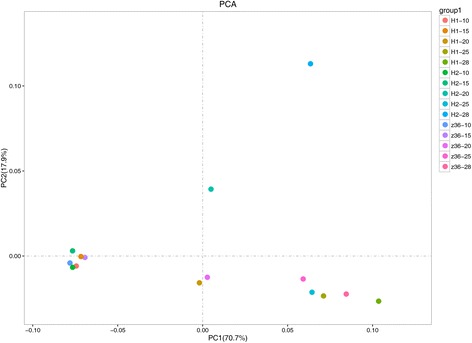
Principal Component Analysis of the genes identified from the 15 samples. H1, H2 and Z36 represent MBI9915, MBI9749 and CCRI36, respectively. 10 = 10 DPA, 15 = 15DPA, 20 = 20DPA, 25 = 25DPA, and 28 = 28DPA

For further confirming the relationship of the three lines at different stages, Principal Component Analysis (PCA) was performed on the above-mentioned expressed genes (Fig. 2). The results indicated that the expressed genes at the same stage in the three lines could be aggregated except for MBI9749 at 20DPA and at 28DPA. The similar gene expression patterns during the same development stage the PCA analysis indirectly proved, to some extent, the reliability of our RNA-seq data.

### Analysis of differentially expressed genes (DEGs)

**Fig. 3:**
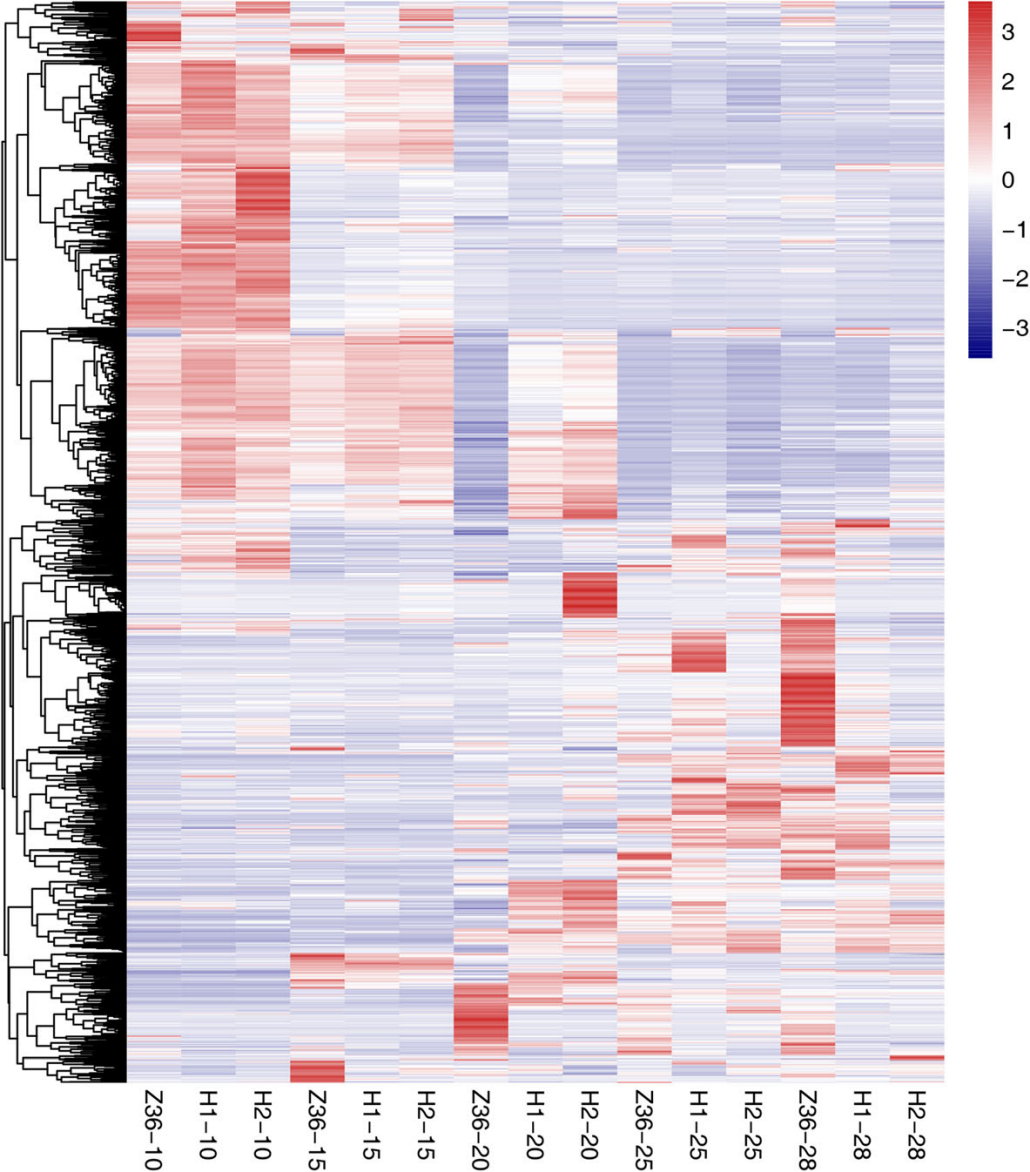
A heat map of 1801 DEGs from 15 samples. H1, H2 and Z36 represent MBI9915, MBI9749 and CCRI36, respectively. 10 = 10 DPA, 15 = 15DPA, 20 = 20DPA, 25 = 25DPA, and 28 = 28DPA

In order to identify DEGs in fiber development, the fragments per kb per million of the mapped reads (FPKM) value was employed to measure the gene expression quantity. The FPKM values were underwent GFOLD (Generalized Fold Change) algorithm to identify DEGs between MBI9915/MBI9749 and CCRI36 at same fiber development stage. Totally 1801 DEGs were obtained, including 898 down-regulated ones and 903 up-regulated ones (Additional file 1). A cluster analysis of the FPKM values of these DEGs in the three lines was carried out, which resulted in a dynamic change of DEGs as shown in a heat map (Fig. 3). Interestingly, a high similarity of expression pattern of DEGs in CCRI36 and the two CSSLs at 10DPA was identified, which was in consistency with the above-mentioned PCC results. From an overall point of view, MBI9915 and MBI9749 had a higher similarity in gene expression profiles which was in contrast to that of CCRI36, indicating that the CSSLs presented a certain degree of difference with respect to their recurrent parent. The dynamically varied DEGs identified from the comparisons among the three lines might reveal a key gene expression regulating mechanism in fiber development and quality formation.

**Fig. 4:**
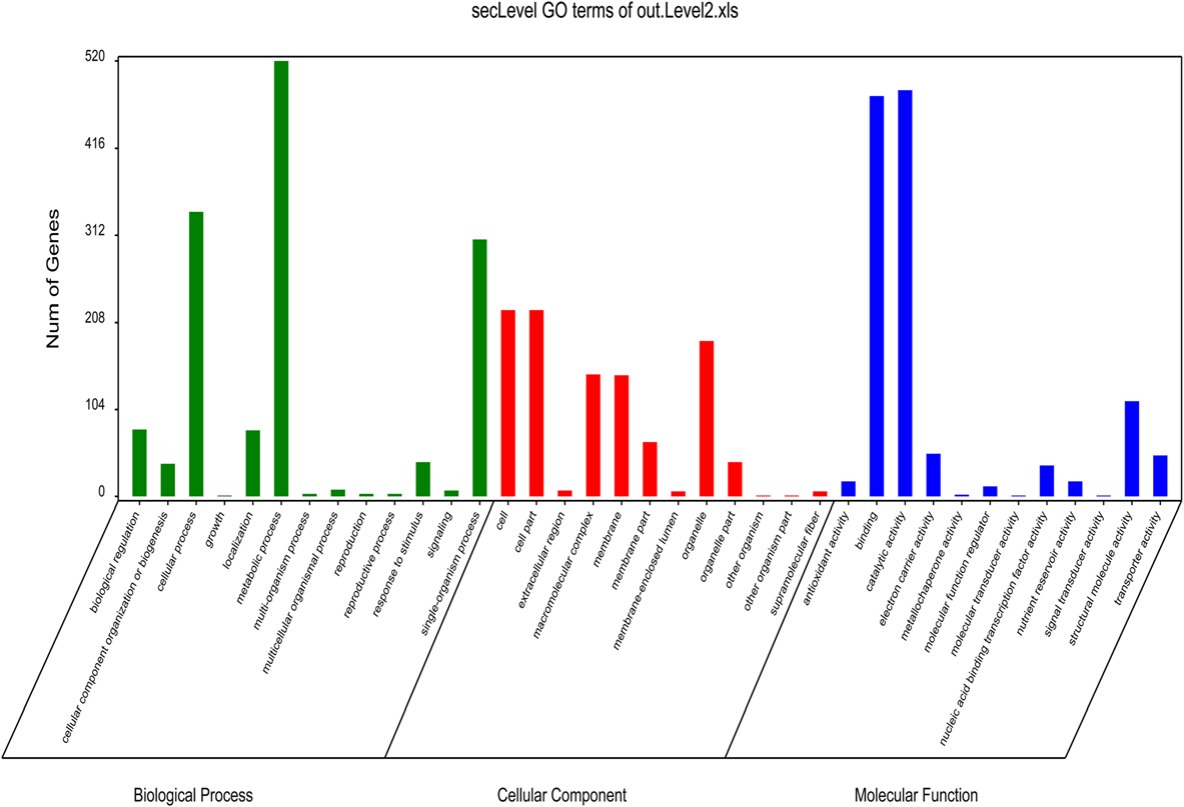
GO functional classification of 1801 DEGs

To comprehend the fiber development processes and identify the putative functional genes, the 1801 DEGs were classified into 37 GO terms based on biological process, cellular component and molecular function (Fig. 4). In the molecular function, most DEGs were sorted into catalytic activity (485 genes, 26.91% of the 1801 DEGs) and binding (478 genes, 26.53%). In the cellular component category, the most abundant subcategories were cell (223 genes, 12.38%) and organelle (186 genes, 10.32%). Under the biological process category, metabolic process (520 genes, 28.86%), cellular process (340 genes, 18.87%) and single-organism process (307 genes, 17.04%) were the most abundant subcategories, while only one gene was categorized into growth process, namely XLOC_018364, which belongs to COBRA-like extracellular glycosyl-phosphatidyl inositol-anchored protein family. According to previous studies, COBRA-Like (COBL) genes encoding a plant-specific glycosylphosphatidylinositol (GPI) anchoring protein have been found to play pivotal roles in the orientation of microfibrils and cellulose crystallinity status, and ultimately make a difference on fiber development and quality formation [55, 56]. Referring to the expression quantity in the current RNA-seq data, XLOC_018364 was found to express throughout all the stages in this study (from 10 to 28DPA) in the three lines in spite of a lower expression level at early stages (10 and 15DPA). While the FPKM value of XLOC_018364 in CCRI36 gradually decreased after reaching the peak at 20DPA, but the maximum FPKM value in MBI9915 and MBI9749 appeared at 28DPA and 25DPA, respectively. Considering the apparent differences between the CSSLs and their recurrent parent, the high activity of COBL gene in the two CSSLs at later developmental stages, might be due to the *G. barbadense* chromosomal segments harbored in them.

**Fig. 5:**
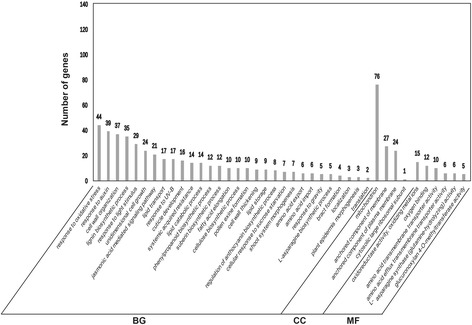
GO enrichment and KEGG pathway analysis of the up-regulated DEGs

**Fig. 6:**
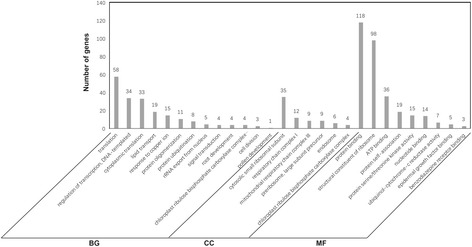
GO enrichment and KEGG pathway analysis of the down-regulated DEGs

For further uncovering the potential functions of these DEGs, GO enrichment and KEGG pathway analysis were respectively performed on the up-regulated and down-regulated genes with Blast2Go software. The 903 up-regulated genes were annotated and categorized into 41 GO terms based on biological process, cellular component and molecular function (Fig. 5). Under the biological processes, most up-regulated DEGs were concentrated on the response to oxidative stress (44 genes, 4.88% of the up-regulated 903 genes), response to auxin (39 genes, 4.32%), cell wall organization (37 genes, 4.10%) and lignin biosynthetic process (35 genes, 3.88%). In the cellular component category, mitochondrion (76 genes, 8.43%) was the most abundant subcategory followed by anchored component of membrane (27 genes, 2.99%) and anchored component of plasma membrane (24 genes, 2.66%). While in molecular function category, oxidoreductase activity and oxidizing metal ions (15 genes, 1.66%), oxygen binding (12 genes, 1.33%) and amino acid transmembrane transporter activity (10 genes, 1.11%) were the principal subcategories. The 898 down-regulated genes were annotated and categorized into 28 GO terms based on biological process, cellular component and molecular function (Fig. 6). The translation (58 genes, 6.46% of the 898 down-regulated genes), regulation of transcription and DNA-templated (34 genes, 3.79%) and cytoplasmic translation (33 genes, 3.67%) enriched most of the down-regulated DEGs under the biological process, while the dominant subcategories in cellular component and molecular function were cytosolic small ribosomal subunit (35 genes, 3.90%), followed by respiratory chain complexI (12 genes, 1.34%) and protein binding (118 genes, 13.14%), structural constituent of ribosome (98 genes, 10.91%), respectively.

**Fig. 7:**
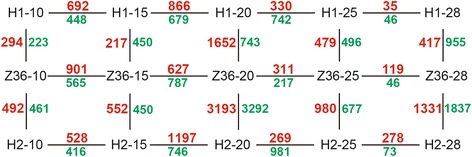
Multiple comparisons between CCRI36 and two CSSLs at diverse stages of fiber development. H1, H2 and Z36 represent MBI9915, MBI9749 and CCRI36, respectively. 10 = 10 DPA, 15 = 15DPA, 20 = 20DPA, 25 = 25DPA, and 28 = 28DPA. Red numbers represent the up-regulated genes, while green ones represent the down-regulated genes

**Fig. 8:**
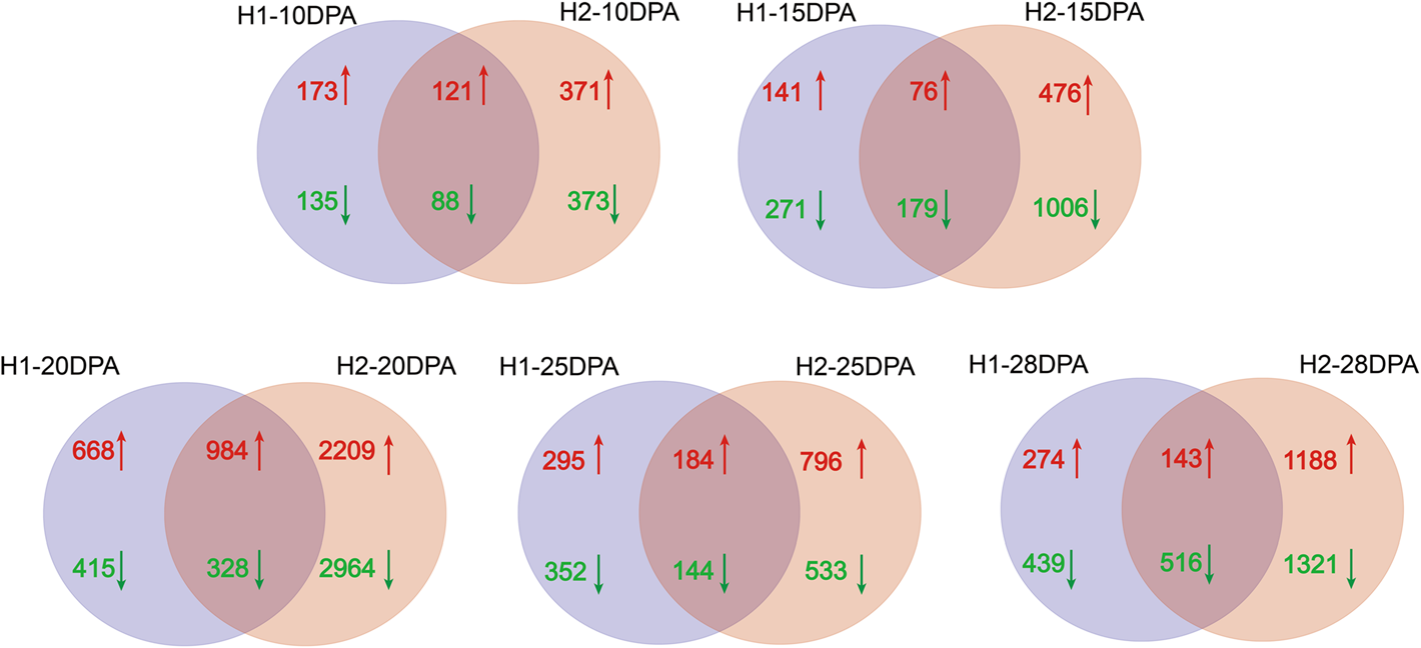
Multiple comparisons of the DEGs between MBI9915 and MBI9749 at the same stage of fiber development. H1 and H2 represent MBI9915 and MBI9749, respectively. 10 = 10DPA, 15 = 15DPA, 20 = 20DPA, 25 = 25DPA and 28 = 28DPA. Red numbers represent the up-regulated genes, while green ones represent the down-regulated genes

To identify the DEGs between CCRI36 and two CSSLs, multiple comparisons among the different fiber developmental stages were performed as shown in Fig. 7. The results indicated that various sets of DEGs were identified between each of the CSSLs and CCRI36. Based on these results, the DEGs of the two CSSLs at each stage were compared and the results were shown in Fig. 8. Totally 209 common DEGs (121 up-regulated and 88 down-regulated) at 10DPA, 255 common DEGs (76 up-regulated and 179 down-regulated) at 15DPA, 1312 common DEGs (984 up-regulated and 328 down-regulated) at 20DPA, 328 DEGs (184 up-regulated and 144 down-regulated) at 25DPA, 659 common DEGs (143 up-regulated and 516 down-regulated) at 28DPA were identified, respectively.

### Analysis of gene temporal expression patterns

For the sake of exploring the temporal patterns of these DEGs in the two CSSLs and CCRI36, the Short Time-series Expression Miner software (STEM) was employed. The results indicated that the expression of these DEGs in all the three lines was assorted into 8 profiles by STEM (Additional file 2, Fig. 9). Each profile represents a set of genes with a similar expression pattern. The largest expression profile of the DEGs in CCRI36 was profile 1 (598 genes, 41.50% of 1441 genes), followed by profile 7 (400 genes, 27.76%), profile 2 (208 genes, 14.43%) and profile 6 (89 genes, 6.18%). However, a similar expression trend was identified in the two CSSLs. In MBI9915, the highest number of DEGs was clustered into profile 1 (685 genes, 50.22% of 1364 genes), followed by profile 7 (282 genes, 20.67%), profile 6 (111 genes, 8.14%) and profile 4 (100 genes, 7.33%). While in MBI9749, the highest number of DEGs was clustered into profile 1 (692 genes, 49.96% of 1385 genes), followed by profile 6 (207 genes, 14.95%), profile 7 (190 genes, 13.72%) and profile 4 (71 genes, 5.13%).

**Fig. 9:**
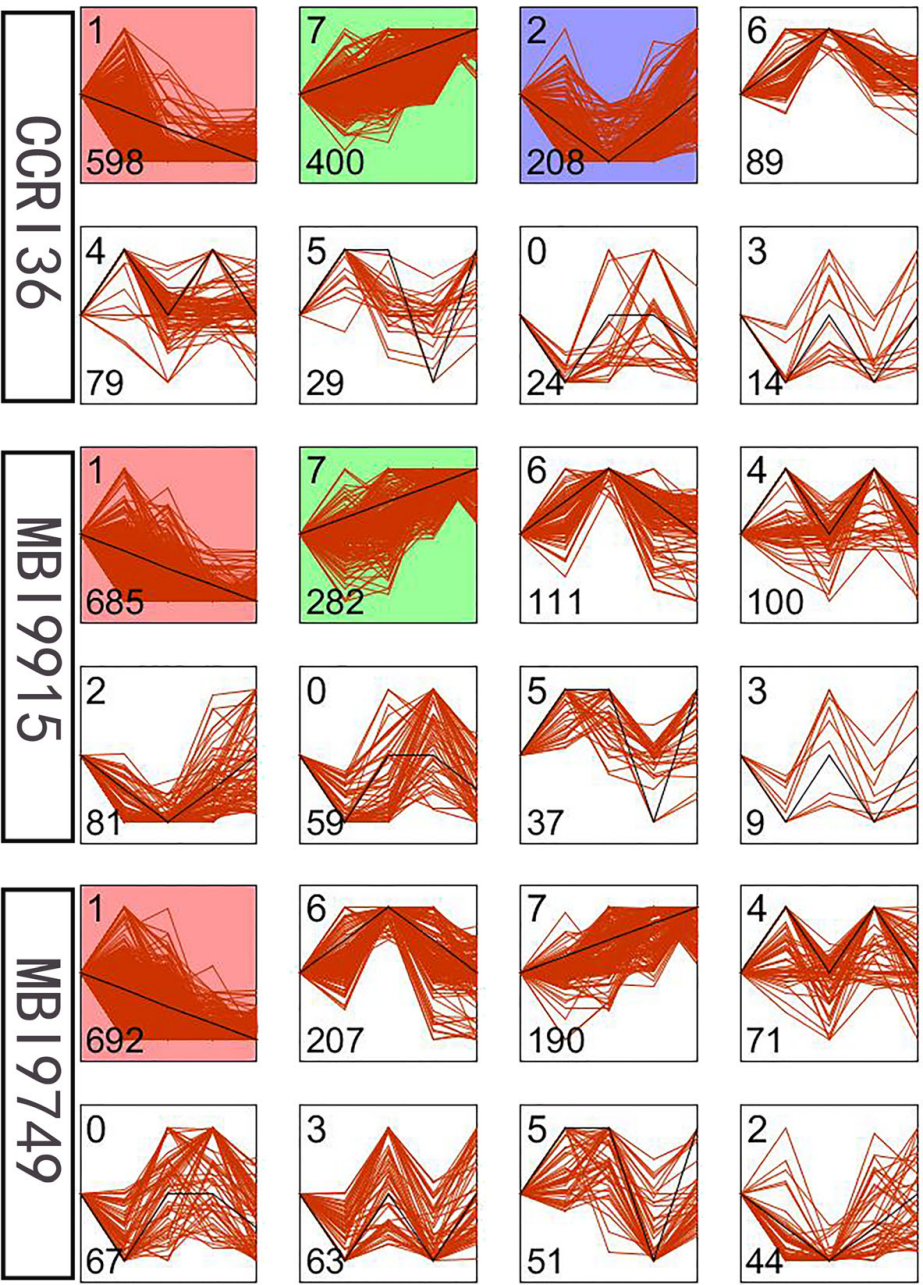
Different gene expression patterns in the three lines. Each square represents a trend of gene expression. The number in top left corner indicates the profile ID number; and the number in bottom left corner indicates the number of genes in that profile. The profiles were ordered based on the number of genes enriched thereof

**Fig. 10:**
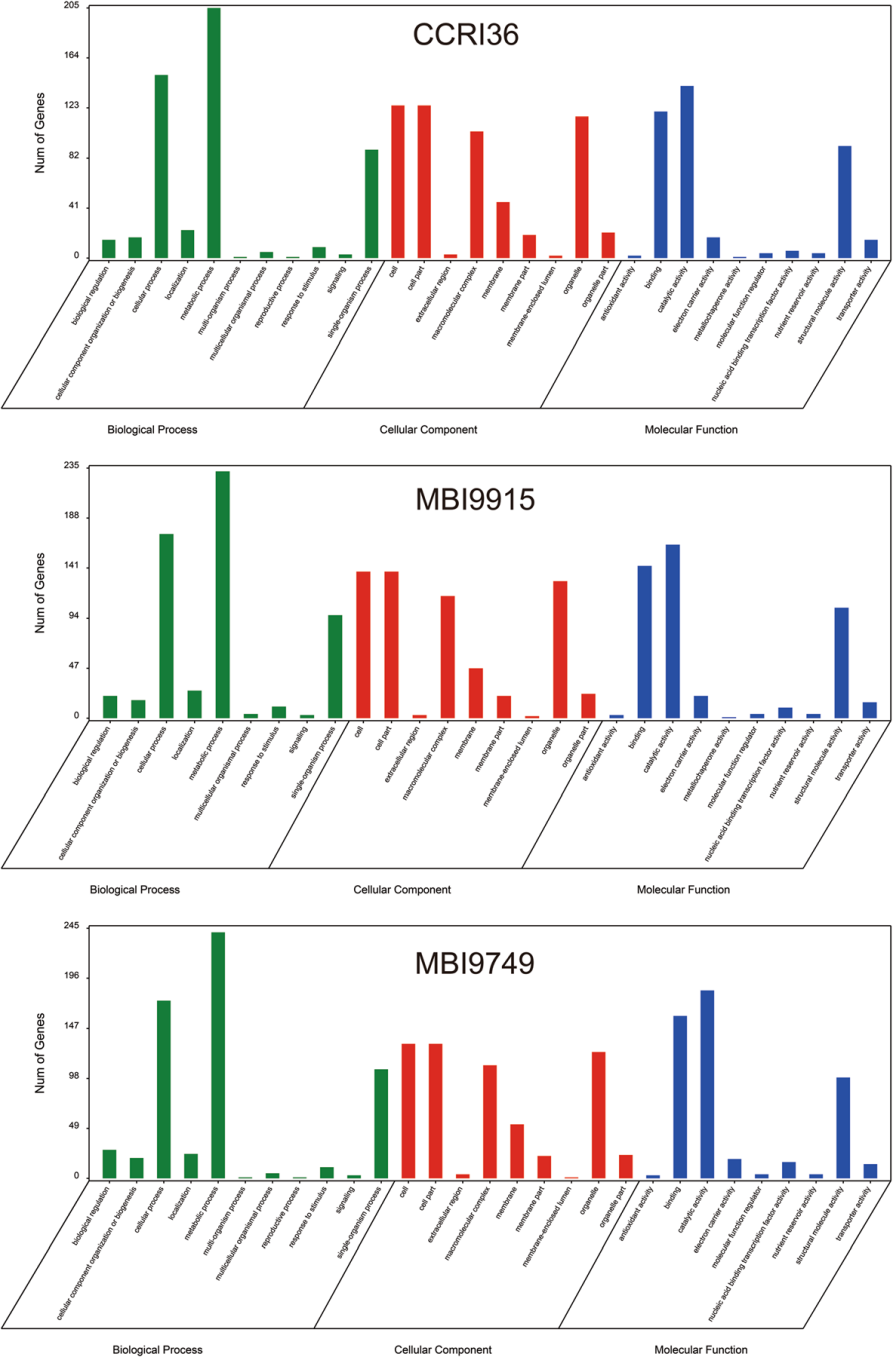
GO functional classification of the genes in profile 1 in the three lines

The DEGs in profile 1 from the three lines were selected to perform a GO-term analysis to try identifying putative functional genes. All the DEGs in profile 1 were classified into 3 categories including biological process, cellular component and molecular function (Fig. 10). In the biological process, metabolic process (200 genes), cellular process (150 genes) and single-organism process (89 genes) were the dominant subcategories in CCRI36. While same trend was identified in the two CSSLs; in MBI9915, 232, 173, 97 DEGs were identified in metabolic process, cellular process and single-organism process respectively and in MBI9749, the same processes were identified to have 241, 174, 107 DEGs, respectively. In the molecular function category, the catalytic activity was the most abundant subcategory in the three lines, while cell was the most abundant subcategory in the cellular component. Based on the above results, the same category/subcategory was identified to have a varied number of DEGs implied that the different gene expression profiles might be responsible for the difference of fiber quality between CCRI36 and the two CSSLs.

**Fig. 11:**
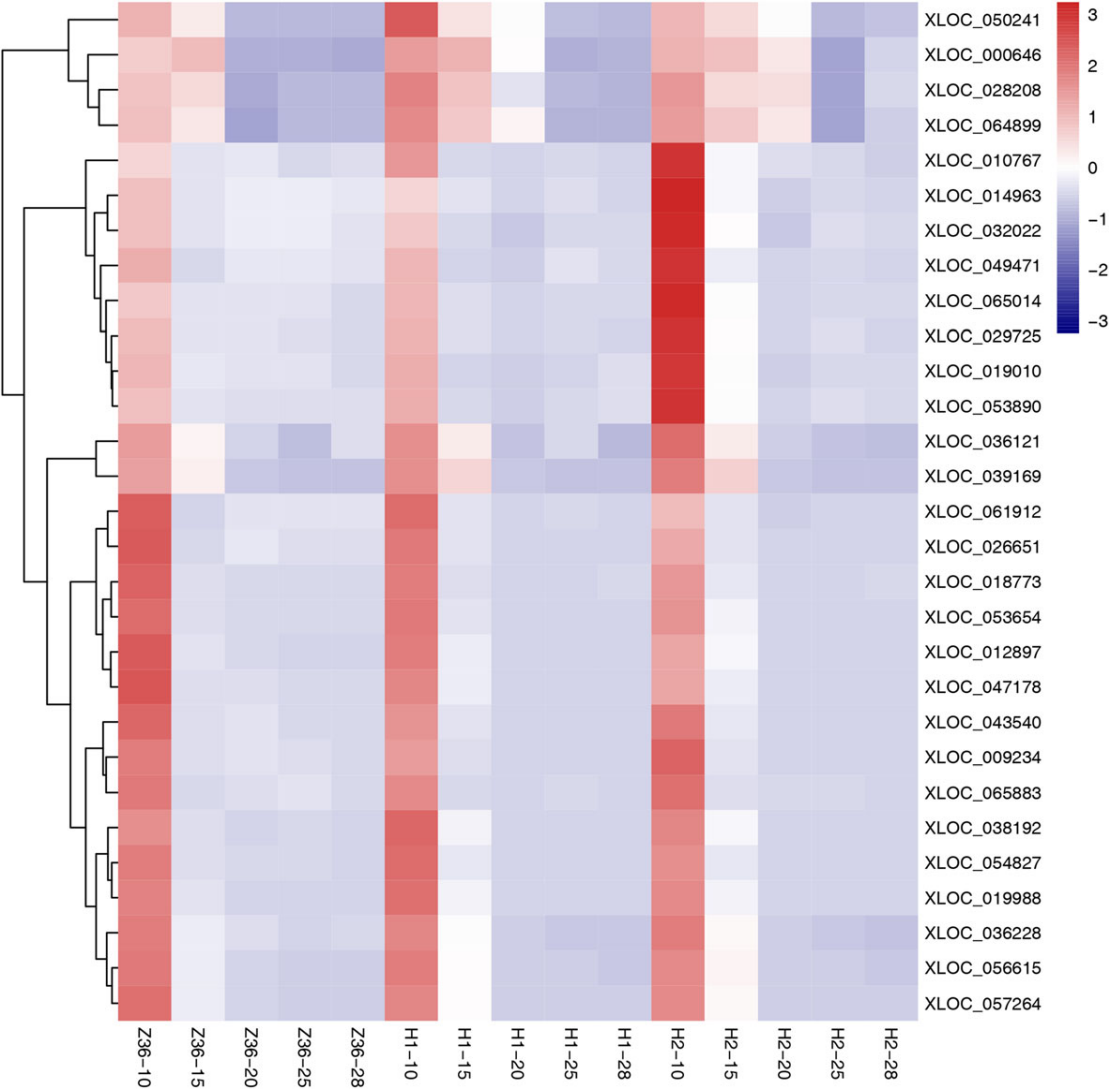
A heat map of the 29 oxidation-reduction process-related genes in profile 1

Instead of above-mentioned processes, there had been increasing evidence that ROS played pivotal roles in fiber cell development along with Ca 2^+^ and actin [57, 58]. As the second messenger during the biological processes, reactive oxygen species (ROS) has been reported to mediate cellular signal transduction and homeostasis regulation by forming the unique biological oxidation and reduction cycle [59], specifically participate in accommodating stress resistance and tip growth including pollen tube growth and root hair elongation in plants [60–64]. Therefore, a total of 29 DEGs in the subcategory “oxidation-reduction process” in biological process were selected to make another cluster analysis based on their FPKM values as shown a heat map (Fig. 11). An expression trend of gradual declining from 10DPA to 28DPA in CCRI36 were identified, while similar tendencies were also observed in MBI9915 and MBI9749 with a timing delay. Currently, 2 peroxidases (XLOC_036121 and XLOC_010767) were identified with high-expression quantity at 10DPA in all the three materials, which is consistent with a previous report that a large accumulation of ROS occurred at the cotton fiber initiation and elongation stage [65]. However, higher expression levels of the two genes were identified in two CSSLs than in CCRI36 at 10DPA, which indicated that fiber cell elongation obtained more enhancements in these genes in the CSSLs, which probably might result in the regulation of the quality formation in the end.

Furthermore, 2 leucoanthocyanidin dioxygenases (XLOC_019010 and XLOC_053890) and 2 flavanone 3-hydroxylases (XLOC_029725 and XLOC_065014) associated with flavonoid pathway were identified in the aforesaid “oxidation-reduction process” subcategory. With strong antioxidant activities, flavonoids, which were relevant to the formation of pigment, participated in plant color alteration and eliminated the reactive oxygen species generated and accumulated under adverse stresses, mainly participated in plant resistance reactions [66] and in the natural coloration of cotton fiber [67]. Some studies reported that flavonoid pathway and its associated genes exhibited remarkable activities in the fiber development of wild and color cotton species [68–71]. Several flavonoid relevant genes in Upland and Sea Island cotton, such as F3H, CHS, ANS and LDOX were found with high level expression during the fiber growth, especially at the early stages of fiber development. Therefore, they were also believed to take part in the control of plant development [72, 73]. Researches indicated that flavonoid pathway might mediate the cotton fiber development with a negative selection in the process of artificial domestication [74, 75]. The four flavonoid genes identified in our RNA-seq results arised with a high-level expression at 10DPA, which is consistent with the above-mentioned research findings, indicating flavonoid pathway might participate in the fiber development, especially at the early stages.

### DEG validation by qRT-PCR

**Fig. 12:**
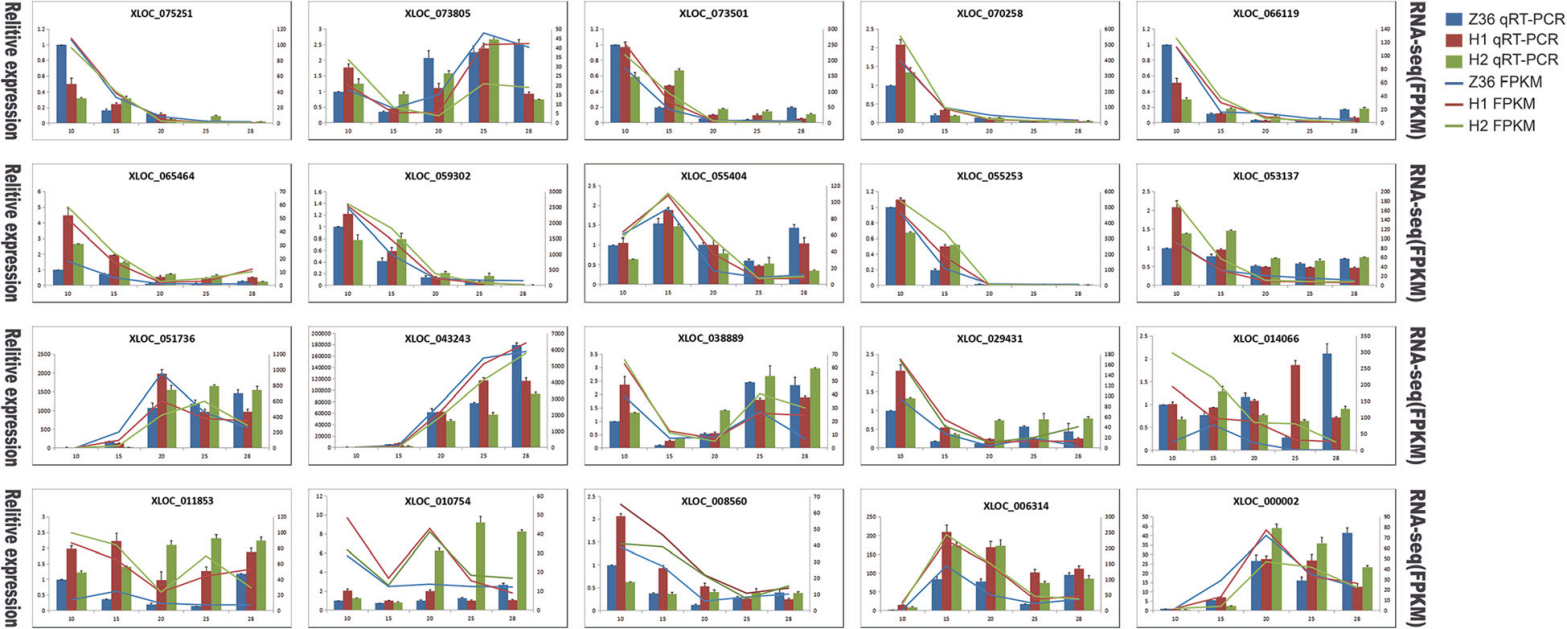
Validation of RNA-seq data by qRT-PCR. H1, H2 and Z36 represent MBI9915, MBI9749 and CCRI36 respectively. Columns indicate the results of qRT-PCR, and zigzag lines indicate the results of transcriptome sequencing

After GO term analysis and KEGG annotation, twenty DEGs, including the ones involved in Starch and sucrose metabolism (XLOC_073805, XLOC_051736), Flavonoid biosynthesis (XLOC_070258, XLOC_053137) and Pentose and glucuronate interconversions (XLOC_055253), were randomly selected to confirm the validity of RNA-seq data (Table 4). The house-keeping gene β-Actin was selected as the reference gene during the validation analysis. The results indicated that expression patterns of 17 genes (85% of 20 genes) were highly consistent with the RNA-seq data, while the remained 3 genes (15%) were partially consistent (Table 4, Fig. 12). These results highly confirmed the reliability of the RNA-seq results.

**Table 4:** Primers used for qRT-PCR validation

Gene ID	Gene name	Forward 5′-3′	Reverse 5′-3′
XLOC_075251	Remorin family	GCGGAGGAAACGAAGAAGGA	TCAGGGGCTTTCTCAACGAC
XLOC_073805	SBE2.1	TTGATTCTGCCCTCCGACAG	GTTGGAACCATCCCATCCGT
XLOC_073501	LTP6	CTCTACGGCGAGGTTCAGAC	GCACCTTGTTGCAGTCAGTG
XLOC_070258	germin 3	ACTTGAATGGCCCTGTAGGC	TAACACCCGGGAATTGAGCC
XLOC_066119	C3HC4-type finger family	GAACCCTCTTGTCCCTGTCG	GCAGTGCCAACCATAAGAGC
XLOC_065464	HSP20	GGCAATCGTAGGGGTAGCAG	CTTTCTTGAGGCCAGGGAGG
XLOC_059302	BXL1	CTCAAGTCATCACCACCGCT	CGTAGTTGGCGGCGTATTTG
XLOC_055404	FKBP	AGTTGGTGAAGAAAAAGAGATTG	GATACCCTCATCCCAGCCCT
XLOC_055253	EAP family	CACACCGACTACAAAGCCCT	CTCTCCACTCCCTTGGCTTG
XLOC_053137	CesA4	TCAAAGGGTCCGCTCCAATC	GGCGAGTAAAGGGATCGAGG
XLOC_051736	FLA7	ACGATACTATGCCCTTGCGG	GACTGGCTTGGACGTGTGTA
XLOC_043243	DFRA	TTATGTCGTCCGAGCCACTG	CGGACTCGAAGTCCATAGGC
XLOC_038889	PL family	GCCAGCGTTCATATTGCAGG	TGGTCAACCCAAACATGGCT
XLOC_029431	ABP1	GCCGATCCATAGGCACTCTT	GCAGTGTGAGGCATCAACCA
XLOC_014066	alpha/beta-Hydrolases superfamily	AGGAGCAGTGTTGTTGCTGA	TGAGGCAATTTGCAGCCAGTA
XLOC_011853	PDF1	CGATTTCTGGAGGAGTCACCC	GCCCCAAGTCCATCAGTACG
XLOC_010754	NAD(P)-binding Rossmann-fold superfamily	GACGTTGGCTGAAAAGGCAG	CAGAAAAACATGGGCTCGGC
XLOC_008560	KCS12	ACTGGAATGGGGTGTAGTGC	CACCCGAACGGAACAAACAA
XLOC_006314	GATA-type zinc finger family	ATGCATGGCCTGTGACAAGA	GAGGCCTGCAATAAAGCACC
XLOC_000002	GNS1/SUR4	GCTTCCTCACCCTCACTGTC	ACCCAGGGCCATGATGAAAG

## Conclusion

Multiple comparisons among MBI9915, MBI9749, and CCRI36 during various fiber developmental stages (10DPA to 28DPA) were conducted in the current study. The results showed that less than 4% of the total substituted segments of *G. barbadense* in the two CSSLs were heterozygous, which indicated the eligibility of the CSSLs for our intended studying of transcriptome analysis. A high proportion of 91.93–94.30% of clean RNA-seq reads were mapped to the reference genome of *G. hirsutum* implied a reliable quality of the sequencing result. A total of 10,198 genes were identified and 1801 DEGs were identified through GFOLD analysis, including 898 down-regulated ones and 903 up-regulated ones. The up-regulated DEGs were mainly enriched in response to oxidative stress and auxin, cell wall organization, and lignin biosynthesis processes while the down-regulated ones in the processes of translation, regulation of transcription, DNA-templated, and cytoplasmic translation. Temporal expression patterns analysis of DEGs revealed that they were mainly enriched in profile 1, profile 7 and profile 6. GO-term analysis of DEGs in profile 1 indicated that these DEGs were mainly enriched in subcategories of metabolic process, cellular process, and single-organism process in biological process in all the three lines. Referring to the previous studies and bioinformatics analysis of the genes identified in the oxidation reduction process subcategory revealed that these genes also played important roles in fiber development in CSSLs in our current study. Our study not only offered novel insights into the developmental mechanism of fiber elongation and SCW biosynthesis, but also discussed the potential contribution of substitution segments to fiber quality performance, which undoubtedly is of great value for cotton breeding and genomics researches.

## Methods

### Plant material

**Fig. 13:**
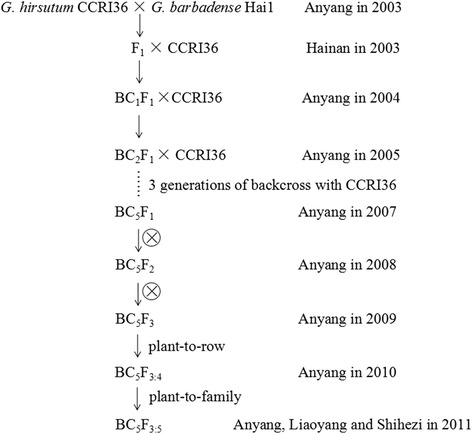
Flow chart of construction of CSSLs

Two Chromosome Segment Substitution Lines (CSSLs) and their *G. hirsutum* recurrent parent CCRI36 planted in the experimental farm of the Institute of Cotton Research, Chinese Academy of Agricultural Sciences (Anyang, Henan Province) were used as plant materials in the current study in 2014. The CSSLs were constructed from a cross between the paternal parent Hai 1 (*G. barbadense*) and the maternal parent CCRI36 (*G. hirsutum*) grown in Anyang in 2003. After five generations of backcrossing with CCRI36 as the recurrent parent and three generations of consecutive selfing, the BC5F3 CSSLs were obtained in 2009. Through plant-to-row method, BC5F3:4 rows were planted in Anyang (Henan province) in 2010 for fiber quality evaluation. In 2011, BC5F3:5 lines were planted in Anyang (Henan province), Liaoyang (Liaoning province) and Shihezi (Xinjing province) for further evaluation. The details of CSSLs construction were shown in Fig. 13.

Based on the high-density linkage map constructed from the aforementioned population of CSSLs [12], 527 Simple Sequence Repeat (SSR) markers were selected to confirm the genetic compositions of CSSLs at an approximate interval of one SSR maker per 10 cM [11]. Owing to the superior fiber performance and relatively clear substituted chromosomal segments composition, MBI9915 and MBI9749 were selected to conduct the current transcriptome study, with their recurrent parent CCRI36 was used as a reference.

Due to concentrating on studying fiber length and strength, our emphasis was laid on the fiber elongation and SCW biosynthesis, which made us to sample the developing fibers at 10, 15, 20, 25 and 28DPA to conduct RNA-seq.

Flowers were tagged on the day of anthesis, which were carried out consecutively for three days as one biological replicate. Three-ten developing bolls from the three lines were harvested by 10:00 a.m. at 10, 15, 20, 25 and 28 day post anthesis (DPA) in each replicate, then immediately immersed in ice. Fiber samples were dissected from the developing bolls, frozen into liquid nitrogen, and stored at −80 °C, which were divided into two portions, one for RNA-seq and the other for qRT-PCR. Totally three replicates were tagged and sampled.

### RNA extraction, library construction and transcriptome sequencing

High-quality RNA extraction was performed from frozen fiber tissue using the RNAprep Pure Plant Kit (Tiangen, Beijing, China). RNA degradation and contamination was confirmed through 1% agarose gel electrophoresis. After being assessed the RNA integrity by the RNA Nano 6000 Assay Kit of the Bioanalyzer 2100 system (Agilent Technologies, CA, USA), RNA concentration was calibrated by Qubit® RNA Assay Kit in Qubit® 2.0 Flurometer (Life Technologies, CA, USA). For minimizing experimental errors, after calibration, RNA samples extracted from the three biological replicates of each stage were mixed with same quantity and volume to generate one sample and were calibrated again for transcriptome sequencing in this study. A total amount of 3 μg RNA sample was used for transcriptome library sequencing, which was constructed using NEBNext® Ultra™ RNA Library rep Kit for Illumina® (NEB, USA) following manufacturer’s recommendations. Firstly, mRNA purification and fragmentation were respectively carried out by poly-T oligo-attached magnetic beads and divalent cations under elevated temperature (94 °C for 5 min) in NEBNext First Strand Synthesis Reaction Buffer (5×). Next, the first strand cDNA synthesis was accomplished using random hexamer primer and M-MuLV Reverse Transcriptase (RNase H-) and the second strand cDNA was synthesized using DNA Polymerase I and RNase H. After purification with AMPure XP system (Beckman Coulter, Beverly, USA), the cDNA segments of 150–200 bp in length were utilized to construct the cDNA libraries through end-repair, adaptor ligation and PCR amplification. At last, the AMPure XP system and Agilent Bioanalyzer 2100 system were separately used to purify the PCR products and to evaluate the library quality. In total, 15 cDNA libraries of 3 cotton lines with 5 stages (10, 15, 20, 25, 28DPA) were sequenced on the Illumina HiSeq™ 2000 sequencing platform.

### Data quality control and reads mapping to the reference genome

Through Base Calling, the raw sequence data were firstly transformed to Sequenced Reads, including reads sequences and corresponding base quality. The raw reads in FASTQ format were subsequently processed by the Illumina pipeline to obtain the clean data by filtering all low-quality reads, such as the reads containing only adapter or poly-N whilst the Q30 and GC contents were calculated. All the downstream analyses were based on the clean data with high quality.

The clean reads were mapped to the *G. hirsutum* reference genome [27], together with gene model annotation files, which was downloaded from the CottonGen database (http://www.cottongen.org). Bowtie v2.0.6 and TopHat v2.0.9 was respectively applied to build the index of the reference genome and to make the reads alignment [76].

### Differentially expressed genes analysis

In order to comprehend the status of differential gene expression in the developmental processes of cotton fiber, 15 separate libraries were established from 5 fiber development stages (10, 15, 20, 25, 28DPA) in the 3 lines, thereby producing comparison groups of the different stages of the same line and the different lines of the same stage. Gene expression levels were subject to transcript abundance, which was calculated as the reads mapped to reference genes or exons. The FPKM (fragments per kb per million of the mapped reads) parameter was employed to count the transcript data [77], whilst the algorithm GFOLD (Generalized Fold Change) was adopted to produce biological meaning and the rankings of DEGs [78]. As the recurrent parent of MBI9915 and MBI9749, CCRI36 was used to compare the two CSSLs to generate GFOLD value. The genes that their absolute GFOLD value >1 was defined as DEGs. Furthermore, Gene Ontology (GO) enrichment analysis of DEGs was performed by Blast2go software with corrected *P*-value ≤ 0.5 as the cutoff. KEGG pathways of DEGs was tested by KOBAS software [79, 80].

### Comparison of expression patterns of DEGs

The Short Time-series Expression Miner software (Carnegie Mellon University, USA) was adopted to identify the temporal expression patterns of genes along with the growth stages of cotton fiber. GO enrichment analysis was also employed to identify the potential functional genes or to characterize the expression profiles identified by STEM analysis.

### Validation of RNA-seq by qRT-PCR

qRT-PCR was employed to verify the veracity of the transcriptome data with twenty randomly selected DEGs. The specific primers of these DEGs were designed by Primer-BLAST of online tool of NCBI. RNA samples were prepared as described in “RNA extraction, library construction and Transcriptome sequencing” section. The cDNAs synthesized using a TranScript All-in-One First-Strand cDNA Synthesis SuperMix for qPCR Kit (Transgen Biotech, Beijing, China). qRT-PCR was performed following the protocol of TransStart Top Green qPCR SuperMix kit (Transgen Biotech, Beijing, China), on the ABI 7500 fast Real-Time PCR System (Applied Biosystems, USA). The housekeeping gene of β-Actin gene was used as the reference to normalize the relative expression levels, with its primer sequences: F: 5′-ATCCTCCGTCTTGACCTTG-3′, and R: 5′-TGTCCGTCAGGCAACTCAT-3′. The qRT-PCR carried out in 20 μL system at the following conditions: one cycle of 94 °C for 30s; 40 cycles of 94 °C for 5 s, 60 °C for 34 s, and one cycle of 60 °C for 60s. Three biological and technical replicates were performed to validate the results in qRT-PCR tests. The relative gene expression level was quantified by the 2^-ΔΔCt^ method [81].

## Additional files

Additional file 1: DEGs in the three lines. (XLS 1648 kb)

Additional file 2: Temporal expression of DEGs in three lines. (XLS 190 kb)

## Abbreviations

ABP1: Auxin binding protein 1
BW: Boll weight
BXL1: Beta-xylosidase 1
CesA4: Cellulose synthase 4
COBLs: COBRA-Like genes
CSSLs: Chromosome segment substitution lines
DEGs: Differentially expressed genes
DFRA: Dihydroflavonol 4-reductase
DPA: Days post anthesis
EAP family: Eukaryotic aspartyl protease family protein
FE: Fiber elongation
FKBP: FKBP-type peptidyl-prolyl cis-trans isomerase family protein
FL: Fiber length
FLA7: FASCICLIN-like arabinoogalactan 7
FM: Fiber micronaire
FPKM: Fragments per Kb per million of the mapped reads
FS: Fiber strength
FU: Fiber uniformity
GFOLD: Generalized fold change
GNS1/SUR4: GNS1/SUR4 membrane protein family
GO: Gene ontology
GPI: Glycosylphosphatidylinositol
HSP20: HSP20-like chaperones superfamily protein
KCS12: 3-ketoacyl-CoA synthase 12
KEGG: Kyoto encyclopedia of genes and genomes
LP: Lint percentage
LTP6: Lipid transfer protein 6
NCBI: National Center for Biotechnology Information
PAGE: Polyacrylamide gel electrophoresis
PCA: Principal component analysis
PCC: Pearson correlation coefficient
PDF1: Protodermal factor 1
PL family: Pectate lyase family protein
qRT-PCR: Quantitative real-time PCR
QTL: Quantitative trait locus
RNA-seq: RNA sequencing
ROS: Reactive oxygen species
SBE2.1: Starch branching enzyme 2.1
SCW: Secondary cell wall
SSR: Simple sequence repeat
STEM: Short time-series expression miner

## References

[1] Wendel J, Albert VA (1992). Phylogenetics of the cotton genus (*Gossypium*): characteristic weighted parsimony analysis of chloroplast-DNA restriction site data and its systematic and biogeographic implications. Syst Bot.

[2] Wendl JF, Brubaker C, Alvarez I, Cronn R, Stewart JM, Paterson AH (2009). Evolution and natural history of the cotton genus. Genetics and genomics of cotton.

[3] Zhang JF, Percy RG, McCarty JC (2014). Introgression genetics and breeding between Upland and Pima cotton: a review. Euphytica.

[4] Wang P, Ding YZ, Lu QX, Guo WZ, Zhang TZ (2008). Development of *Gossypium barbadense* chromosome segment substitution lines in the genetic standard line TM-1 of *Gossypium hirsutum*. Chin Sci Bull.

[5] Lacape JM, Llewellyn D, Jacobs J, Arioli T, Becker D, Calhoun S (2010). Meta-analysis of cotton fiber quality QTLs across diverse environments in a *Gossypium hirsutum* × *G. barbadense* RIL population. BMC Plant Biol.

[6] Yu JZ, Ulloa M, Hoffman SM, Kohel RJ, Pepper AE, Fang DD (2014). Mapping genomic loci for cotton plant architecture, yield components, and fiber properties in an interspecific (*Gossypium hirsutum* L. × *G. barbadense* L.) RIL population. Mol Gen Genomics.

[7] Said JI, Song M, Wang H, Lin Z, Zhang X, Fang DD (2015). A comparative meta-analysis of QTL between intraspecific *Gossypium hirsutum* and interspecific *G. hirsutum* × *G. barbadense* populations. Mol Gen Genomics.

[8] Said JI, Knapka JA, Song M, Zhang J (2015). Cotton QTLdb: a cotton QTL database for QTL analysis, visualization, and comparison between *Gossypium hirsutum* and *G. hirsutum* × *G. barbadense* populations. Mol Gen Genomics.

[9] Wu M, Zhang L, Li X, Xie X, Pei W, Yu J (2016). A comparative transcriptome analysis of two sets of backcross inbred lines differing in lint-yield derived from a *Gossypium hirsutum* × *Gossypium barbadense* population. Mol Gen Genomics.

[10] Zheng JY, Oluoch G, Riaz Khan MK, Wang XX, Cai XY, Zhou ZL, et al. Mapping QTLs for drought tolerance in an F2:3 population from an inter-specific cross between *Gossypium tomentosum* and *Gossypium hirsutum*. Genet Mol Res. 2016;15(3):gmr.15038477. doi:10.4238/gmr.15038477. 10.4238/gmr.15038477 27525919

[11] Zhai H, Gong W, Tan Y, Liu A, Song W, Li J, et al. Identification of chromosome segment substitution lines of *Gossypium barbadense* substituted in *G. hirsutum* and quantitative trait locus mapping for fiber quality and yield traits. PLoS One. 2016;11(9):e0159101. doi:10.1371/journal.pone.0159101. 10.1371/journal.pone.0159101 PMC5014324 27603312

[12] Shi Y, Li W, Li A, Ge R, Zhang B, Li J (2015). Constructing a high-density linkage map for *Gossypium hirsutum* × *Gossypium barbadense* and identifying QTLs for lint percentage. J Integr Plant Biol.

[13] Shi Y, Zhang B, Liu A, Li W, Li J, Lu Q (2016). Quantitative trait loci analysis of *Verticillium* wilt resistance in interspecific backcross population of *Gossypium hirsutum* × *Gossypium barbadense*. BMC Genomics.

[14] Eshed Y, Zamir D (1995). An introgression line population of *Lycopersicon Pennellii* in the cultivated tomato enables the identification and fine mapping of yield-associated QTL. Genetics.

[15] Liu SB, Zhou RG, Dong YC, Li P, Jia JZ (2006). Development, utilization of introgression lines using a synthetic wheat as donor. Theor Appl Genet.

[16] Takai T, Nonoue Y, Yamamoto SI, Yamanouchi U, Matsubara K, Liang ZW (2007). Development of chromosome segment substitution lines derived from backcross between indica donor rice cultivar ‘ Nona bokra ’ and japonica recipient cultivar ‘ Koshihikari ’. Breed Sci.

[17] Zhu W, Lin J, Yang D, Zhao L, Zhang Y, Zhu Z (2009). Development of chromosome segment substitution lines derived from backcross between two sequenced rice cultivars, *Indica* recipient 93-11 and *Japonica* donor nipponbare. Plant Mol Biol Report.

[18] Basara AS, Malik CP (1984). Development of cotton fiber. Int Rev Cytol.

[19] Wilkins TA, Jernstedt JA, Basra AS (1999). Molecular genetics of developing cotton fibers. Cotton fibers: developmental biology, quality improvement, and textile processing.

[20] Kim HJ, Triplett BA (2001). Cotton fiber growth in planta and in vitro. Models for plant cell elongation and cell wall biogenesis. Plant Physiol.

[21] Lee JJ, Hassan OS, Gao W, Wei NE, Kohel RJ, Chen XY (2006). Developmental and gene expression analyses of a cotton naked seed mutant. Planta.

[22] Lee JJ, Woodward AW, Chen ZJ (2007). Gene expression changes and early events in cotton fiber development. Ann Bot-London.

[23] Schubert AM, Benedict CR, Berlin JD (1973). Cotton fiber development-kinetics of cell elongation and secondary wall thickening. Crop Sci.

[24] Wang K, Wang Z, Li F, Ye W, Wang J, Song G (2012). The draft genome of a diploid cotton *Gossypuim raimondii*. Nat Genet.

[25] Li F, Fan G, Wang K, Sun F, Yuan Y, Song G (2014). Genome sequence of the cultivated cotton *Gossypium arboreum*. Nat Genet.

[26] Li F, Fan G, Lu C, Xiao G, Zou C, Kohel RJ (2015). Genome sequence of cultivated Upland cotton (*Gossypium hirsutum* TM-1) proviades insights into genoem evolution. Nat Biotechnol.

[27] Zhang T, Hu Y, Jiang W, Fang L, Guan X, Chen J (2015). Sequencing of allotertraploid cotton (*Gossypium hirsutum* L. acc. TM-1) provides a resource for fiber improvement. Nat Biotechenol.

[28] Yuan D, Tang Z, Wang M, Gao W, Tu L, Jin X (2015). The genome sequence of Sea-Island cotton (*Gossypium barbadense*) provides insights into the allopolyploidization and development of superior spinnable fibers. Sci Rep.

[29] Liu X, Zhao B, Zheng HJ, Hu Y, Lu G, Yang CQ (2015). *Gossypium barbadense* genome sequence provides insight into the evolution of extra-long staple fiber and specialized metabolites. Sci Rep.

[30] Hinchliffe DJ, Meredith WR, Yeater KM, Kim HJ, Woodward AW, Chen ZJ (2010). Near-isogenic cotton germplasm lines that differ in fiber-bundle strength have temporal differences in fiber gene expression patterns as revealed by comparative high-throughput profiling. Theor Appl Genet.

[31] Pang Y, Wang H, Song WQ, Zhu YX (2010). The cotton ATP synthase δ1 subunit is required to maintain a higher ATP/ADP ratio that facilitates rapid fiber cell elongation. Plant Biol (Stuttg).

[32] Lacape JM, Claverie M, Vidal RO, Carazzolle MF, Guimarães Pereira GA, Ruiz M, et al. Deep sequencing reveals differences in the transcriptional landscapes of fibers from two cultivated species of cotton. PLoS One. 2012;7(11):e48855. doi:10.1371/journal.pone.0048855. 10.1371/journal.pone.0048855 PMC3499527 23166598

[33] Runavot JL, Guo X, Willats WG, Knox JP, Goubet F, Meulewaeter F. Non-cellulosic polysaccharides from cotton fiber are differently impacted by textile processing. PLoS One. 2014;9(12):e115150. doi:10.1371/journal.pone.0115150. 10.1371/journal.pone.0115150 PMC4269390 25517975

[34] Yoo MJ, Wendel JF. Comparative evolutionary and developmental dynamics of the cotton (*Gossypium hirsutum*) fiber transcriptome. PLoS Genet. 2014;10:e1004073. doi:10.1371/journal.Pgen.1004073. 10.1371/journal.pgen.1004073 PMC3879233 24391525

[35] Gong W, He S, Tian J, Sun J, Pan Z, Jia Y, et al. Comparison of the transcriptome between two cotton lines of different fiber color and quality. PLoS One. 2014;9(11):e112966. doi:10.1371/journal.pone.0112966. 10.1371/journal.pone.0112966 PMC4234635 25401744

[36] Beasley CA, Ting IP (1974). The effects of plant growth substances on in vitro fiber development from unfertilized cotton ovules. Am J Bot.

[37] Guinn G, Brummett DL (1988). Changes in abscisic acid and indoleacetic acid before and after anthesis relative to changes in abscission rates of cotton fruiting forms. Plant Physiol.

[38] Gokani SJ, Thaker VS (2002). Role of gibberellic acid in cotton fiber development. J Agric Sci.

[39] Sun Y, Fokar M, Asami T, Yoshida S, Allen RD (2004). Characterization of the brassinosteroid insensitive 1 genes of cotton. Plant Mol Biol.

[40] Shi YH, Zhu SW, Mao XZ, Feng JX, Qin YM, Zhang L (2006). Transcriptome profiling, molecular, biological and physiological studies reveal a major role for ethyene in cotton fiber cell elongation. Plant Cell.

[41] Guinn G (1982). Fruit age and changes in abscisic acid content, ethtlene production and abscission rate of cotton fruits. Plant Physiol.

[42] Davis LA, Addicott FT (1972). Abscisic acid: correlations with abscission and with development in the cotton fruit. Plant Physiol.

[43] Gokani SJ, Kumar R, Thaker VS (1998). Potential role of abscisic acid in cotton fiber and ovule development. Plant Growth Regul.

[44] Yang YM, Xu CN, Wang BM, Jia JZ (2001). Effects of plant growth regulators on secondary wall thickening of cotton fibers. Plant Growth Regul.

[45] Chen JG, Du XM, Zhou X, Zhao HY (1997). Levels of cytokinins in the ovules of cotton mutants with altered fiber development. Plant Growth Regul.

[46] Liu K, Sun J, Yao LY, Yuan YL (2012). Transcriptome analysis reveals critical genes and key pathways for early cotton fiber elongation in Ligon lintless-1 mutant. Genomics.

[47] Guo JY, Wang LJ, Chen SP, Hu WL, Chen XY (2007). Gene expression and metabolite profiles of cotton fiber during cell elongation and secondary cell wall synthesis. Cell Res.

[48] Pang CY, Wang H, Pang Y, Xu C, Jiao Y, Qin YM (2010). Comparative proteomics indicates that biosynthesis of pectic precursors is important for cotton fiber and *Arabidopsis* root hair elongation. Mol Cell Proteomics.

[49] Hua SJ, Wang XD, Yuan SN, Shao MY, Zhao XQ, Zhu SJ (2007). Characterization of pigmentation and cleeulose synthesis in colored cotton fibers. Crop Sci.

[50] Han LB, Li YB, Wang HY, Wu XM, Li CL, Luo M (2013). The dual functions of WLIM1a in cell elongation and secondary wall formation in developing cotton fibers. Plant Cell.

[51] Qin YM, Pujol FM, Hu CY, Feng JX, Kastaniotis AJ, Hilttunen JK (2007). Genetic and biochemical studies in yeast reveal that the cotton fiber-specific *GhCER6* gene function in fatty acid elongation. J Exp Bot.

[52] Li Y, Tu L, Ye Z, Wang M, Gao W, Zhang X (2015). A cotton fiber-preferential promoter, PGbEXPA2, is regulated by GA and ABA in *Abrabidopsis*. Plant Cell Rep.

[53] Li Y, Tu L, Pettolino FA, Ji S, Hao J, Yuan D (2016). *GbEXPATR*, a species-specific expansin, enhances cotton fibre elongation through cell wall restructuring. Plant Biotechnol J.

[54] Zhang F, Jin X, Wang L, Li S, Wu S, Cheng C (2016). A cotton annexin affects elongation and secondary Cell Wall biosynthesis associated with Ca 2+ Influx, ROS Homeostasis, and Actin Filament Reorganization. Plant Physiol.

[55] Roudier F, Fernandez AG, Fujita M, Himmelspach R, Borner GH, Schindelman G (2015). COBRA, an Arabidopsis extracellular glycosyl-phosphatidyl inositol-anchored protein, specifically controls highly anisotropic expansion through its involvement in cellulosemicrofibril orientation. Plant Cell.

[56] Niu E, Shang X, Cheng C, Bao J, Zeng Y, Cai C, et al. Comprehensive analysis of COBRA-Like (COBL) gene family in Gossypium identifies two COBLs potentially associated with fiber quality. PLoS One. 2015;10(12):e0145725.doi:10.1371/journal.pone.0145725. 10.1371/journal.pone.0145725 PMC4692504 26710066

[57] Qin YM, Zhu YX (2011). How cotton fibers elongate: a tale of linear cell- growth mode. Curr Opin Plant Biol.

[58] Tang W, Tu L, Yang X, Tan J, Deng F, Hao J (2014). The calcium sensor GhCaM7 promotes cotton fiber elongation by modulating reactive oxygen species (ROS) production. New Phytol.

[59] Dickinson BC, Chang CJ (2011). Chemistry and biology of reactive oxygen species in signaling or stress responses. Nat Chem Biol.

[60] Zhou H, Liu X, Liu L, Yang Z, Zhang S, Tang M (2009). Oxidative stress and apoptosis of human brain microvascular endothelial cells induced by free fatty acids. J Int Med Res.

[61] Potocký M, Jones MA, Bezvoda R, Smirnoff N, Zárský V (2007). Reactive oxygen species produced by NADPH oxidase are involved in pollen tube growth. New Phytol.

[62] Kudla J, Batistic O, Hashimoto K (2010). Calcium Signals: the lead currency of plant information processing. Plant Cell.

[63] Vazquez LA, Sanchez R, Hernandez-Barrera A, Zepeda-Jazo I, Sanchez F, Quinto C (2014). Actin polymerization drives polar growth in *Arabidopsis* root hair cells. Plant Signal Behav.

[64] Mei W, Qin Y, Song W, Li J, Zhu Y (2009). Cotton *GhPOX1* encoding plant class III peroxidase may be responsible for the high level of reactive oxygen species production that is related to cotton fiber elongation. J Genet Genomics.

[65] Di Ferdinando M, Brunetti C, Fini A, Tattini M, Ahmad P, Prasad MNV (2012). Flavonoids as antioxidants in plants under abiotic stresses. Abiotic stress responses in plants.

[66] Murphy A, Peer WA, Taiz Ferdinando L (2000). Regulation of auxin transport by aminopeptidases and endogenous flavonoids. Planta.

[67] Xiao YH, Zhang ZS, Yin MH, Luo M, Li XB, Hou L (2007). Cotton flavonoid structural genes related to the pigmentation in brown fibers. Biochem Biophys Res Commun.

[68] Feng H, Tian X, Liu Y, Li Y, Jones BJ, Sun Y (2013). Analysis of flavonoids and the flavonoid structural genes in brown fiber of upland cotton. PLoS One.

[69] Tan J, Tu L, Deng F, Hu H, Nie Y, Zhang X (2013). A genetic and metabolic analysis revealed that cotton fiber cell development was retarded by flavonoid naringenin. Plant Physiol.

[70] Xiao YH, Yan Q, Ding H, Luo M, Hou L, Zhang M (2014). Transcriptome and biochemical analyses revealed a detailed proanthocyanidin biosynthesis pathway in brown cotton fiber. PLoS One.

[71] Buer CS, Muday GK (2004). The transparent testa4 mutation prevents flavonoid synthesis and alters auxin transport and the response of Arabidopsis roots to gravity and light. Plant Cell.

[72] Shirley BW, Kubasek WL, Storz G, Bruggemann E, Koornneef M, Ausubel FM (1995). Analysis of Arabidopsis mutants deficient in flavonoid biosynthesis. Plant J.

[73] Haughn G, Chaudhury A (2005). Genetic analysis of seed coat development in Arabidopsis. Trends Plant Sci.

[74] Tu LL, Zhang XL, Liang SG, Feng JX, Qin YM, Zhang L (2007). Genes expression analyses of sea-island cotton (Gossypium barbadense L.) during fiber development. Plant Cell Rep.

[75] Ji SJ, Lu YC, Feng JX, Wei G, Li S, Shi YH (2003). Isolation and analyses of genes preferentially expressed during early cotton fiber development by subtractive PCR and cDNA array. Nucleic Acids Res.

[76] Kim D, Pertea G, Trapnell C, Pimentel H, Kelley R, Salzberg SL (2013). TopHat2: accurate alignment of transcriptomes in the presence of insertions, deletions and gene fusions. Genome Biol.

[77] Mortazavi A, Schaeffer L, Wold B (2008). Mapping and quantifying mammalian transcriptomes by RNA-Seq. Nat Methods.

[78] Feng J, Meyer CA, Wang Q, Liu JS, Shirley Liu X, Zhang Y (2012). GFOLD: a generalized fold change for ranking differentially expressed genes from RNA-seq data. Bioinformatics.

[79] Conesa A, Götz S. Blast2GO: a comprehensive suite for functional analysis in plant genomics. Int J Plant Genomics. 2008:619832. doi:10.1155/2008/619832. 10.1155/2008/619832 PMC2375974 18483572

[80] Kanehisa M, Sato Y, Kawashima M, Furumichi M, Tanabe M (2016). KEGG as a reference resource for gene and protein annotation. Nucleic Acids Res.

[81] Livaka KJ, Schmittgen TD (2001). Analysis of relative gene expression data using real-time quantitative PCR and the 2^- ΔΔ CT^ method. Methods.

